# Towards the Developing and Designing of an Intervention to Promote Prenatal Physical Activity Using MomConnect (mHealth): A Formative Protocol

**DOI:** 10.3390/mps8020026

**Published:** 2025-03-03

**Authors:** Uchenna Benedine Okafor, Daniel Ter Goon, Rudolph Leon van Niekerk

**Affiliations:** 1 Department of Public Health, University of Fort Hare, 5 Oxford Street, East London 5201, South Africa; 2 Faculty of Health Sciences, University of Limpopo, Sovenga 0727, South Africa; danielgoon23@gmail.com; 3 Department of Psychology, University of Fort Hare, 50 Church Street, East London 5201, South Africa; leonvn@ufh.ac.za

**Keywords:** mHealth technology, MomConnect, physical activity and exercise, pregnancy, South Africa

## Abstract

Background and aim: The use of mHealth, especially short-message text (SMS), has proven to be an effective intervention in promoting behavioral health outcomes in populations across different contexts and settings. While MomConnect, an mHealth technological device designed to enhance maternal and child health services in South Africa, offers various health-related contents aimed at improving maternal outcomes for pregnant and postpartum women, it currently lacks information on prenatal physical activity. However, physical activity and exercise during pregnancy is safe and beneficial for both the mother and the baby. This article outlines the protocol for designing and developing a prenatal physical activity and exercise text messaging content for the MomConnect device. To achieve this, the protocol aims to elucidate the preferences of prenatal physical activity and exercise text messages and ascertain the preferred amount of SMS messaging to inform the design of an intervention for the incorporation of prenatal physical activity and exercise text messages into the MomConnect device in South Africa. Methods: We will apply a user-centred design approach conducted in three phases. First, a scoping literature review and interviews with pregnant women will be conducted to inform the formative stage for developing a desirable prototype SMS. Secondly, healthcare providers and pregnant women will be interviewed to collate data on the preferred SMS. Lastly, a cross-sectional survey will be conducted to determine the preferred quantity of SMS messaging to be incorporated in the MomConnect device. Expected outcomes: A preferred or desirable prenatal physical activity and exercise SMS text message will inform the design of SMS text messages to be incorporated into the content of the MomConnect device to promote prenatal physical activity and exercise participation among women in the Eastern Cape Province. This study will develop a tailored mHealth intervention to improve prenatal physical activity participation and health behaviors among pregnant women in South Africa.

## 1. Introduction

Pregnancy is an important phase in any woman’s life marked that is with unique physiological changes, which can influence lifestyle behaviors, such as physical activity and exercise. Providing pregnant women with adequate maternal health counseling, which includes physical activity and exercise, is crucial for both the mother and the baby. Research has demonstrated that engaging in physical activity during pregnancy improves maternal outcomes. Thus, healthcare providers should advise pregnant women without any medical problems to exercise regularly. Research shows that frequent activity during pregnancy reduces the incidence of prenatal hypertension, diabetes, obesity, pre-eclampsia, and musculoskeletal lower back pain and shortens labor [1,2,3,4]. Also, prenatal physical activity enhances mental health and quality and decreases the risk of postpartum depression [5,6]; improves breastfeeding outcomes [7]; decreases neonatal complications; and improves the fetal nervous system of the child [8,9,10]. Inactivity during pregnancy could negate the benefits of exercise. However, individuals in both developing [11,12,13,14] and developed [15] countries exhibit significantly lower levels of physical activity compared to those in other countries, a trend that is associated with increased sedentary behavior [16,17]. Similarly, in South Africa, earlier studies have reported low prenatal physical activity [18,19]. This highlights the importance of advocacy and counseling to encourage physical activity and exercise during pregnancy in order to avoid sedentary behaviors.

There are studies in the literature on the reasons for inactivity during pregnancy. These include a lack of motivation, a lack of knowledge, and safety concerns [20,21,22,23,24]. Raising awareness about the importance of physical activity during pregnancy might improve women’s attitudes toward prenatal physical activity. As such, exploring feasible interventional strategies, such as digital technology (mHealth), to promote the uptake of physical activity and exercise during pregnancy and achieve desirable maternal and fetal health outcomes is crucial.

Digital behavioral intervention through text messaging could potentially aid in the promotion of prenatal physical activity and confer multiple advantages. Mobile health (mHealth) interventions help recognize the benefits and opportunities of exercise, create attainable goals, and promote accountability [25], and they are widely accessible and cheap to use [26]. Several studies have shown the efficacy of mHealth interventions in promoting PA in varying populations [25,27,28,29]. Notably, motivational text messaging is potentially a positive adjutant to an mHealth PA intervention [30,31]. Nevertheless, the impact of motivational messages on physical activity behavior appears insignificant and declines over time [30]. Therefore, personalizing and adapting physical activity text messages to participants’ contextual characteristics could potentially ensure the sustainability of a desired change in physical activity behavior.

However, scant information exists on the use of text messages to encourage physical activity and exercise during pregnancy in South Africa. Notably, pregnant women and other stakeholders have advocated for the incorporation of physical activity content in the existing mHealth technology (MomConnect) in South Africa to promote prenatal activity participation [32]. In addition, a study on mHealth interventions to improve antenatal care seeking and health behavioral determinants during pregnancy among adolescent girls and young women in South Africa highlights that the participants expressed a desire for the inclusion of exercise activity in the content of MomConnect [33].

MomConnect is one of the popular, phone-based technological maternal initiative health programs designed by the National Department of Health in South Africa to support and improve maternal health and child health services [34,35,36,37]. The program registers expectant mothers into a national pregnancy registry, sends weekly informational texts, and creates an interactive support desk [34,35,36]. MomConnect sends targeted maternal and child health information to mothers during pregnancy and up to one year postpartum [35]. The MomConnect health promotional messages are freely available to users (pregnant women) in all of the 11 South African official languages. The health messages cover topics such as antenatal care, accessing care during labor, diet and nutrition, managing non-pregnancy-related infections, managing hypertension, caring for newborns, breastfeeding, and vaccination. The absence of MomConnect content regarding physical activity and exercise engagement during pregnancy highlights the importance of this study. This protocol is designed to conduct a feasibility study to provide the groundwork for the designing and development of acceptable prenatal physical activity text messages to aid the facilitation of physical activity among pregnant women in the context of the Eastern Cape Province, South Africa, where physical activity participation is reportedly low due to varying factors [18,32]. It is probable that expectant women and mothers who receive prenatal physical-activity-related information via ‘SMSs’ linked to the MomConnect device may be encouraged to engage in physical activity during pregnancy.

### 1.1. Objectives

This protocol paper aims to conduct a literature review on the preferred and acceptable SMS prenatal physical activity among pregnant women, develop and design a prototype prenatal physical activity text message for infusion into the MomConnect device, and determine the frequency and timing of the SMS test messages.

### 1.2. Expected Outcome

The planned study’s findings will guide the design and implementation of a digital prenatal physical activity program that uses SMS text messages incorporated into the MomConnect health device to improve physical activity behavior during pregnancy.

## 2. Methodology

### 2.1. Research Design

Applying the methodological approach used by Huberty et al. [38], this study will employ a user-centred approach to gather qualitative and quantitative data from both pregnant and non-pregnant women with the goal of designing a prototype prenatal physical activity SMS that can be integrated into the MomConnect technological device to encourage active physical activity during pregnancy. The findings from the qualitative data will inform the design of Phase III, which is the quantitative survey study. Table 1 provides a methodological summary of the study protocol, highlighting each phase of the study.

### 2.2. Phase 1: Interviews and Prototype Development

This phase will employ qualitative research methods, including interviews and literature reviews, to explore the needs and opinions of pregnant women regarding their preferred and acceptable prenatal exercise routines. We will conduct interviews with expectant women who participate in moderate-intensity physical activity, as recommended by various bodies and organizations (The Royal Australian New Zealand College of Obstetricians and Gynaecologists [39,40,41,42]), to identify potential themes relating to barriers and strategies for the text message prototypes [38]. Women visiting antenatal clinics will be invited to participate in the study. Active pregnant women and women who have been previously pregnant, 18 years or older, able to speak English and isiXhosa, and engaged in at least 30 min of moderate activity five days a week (more than 150 min) in the preceding week (self-reported) [38] will be purposively selected to participate in the study.

Participants who agree to participate in the study will be requested to confirm their eligibility in person before an interview is scheduled. With permission from the selected health facility managers, the participants will be interviewed in a quiet designated place provided in each of the health facilities before antenatal sessions. All interviews will be recorded and transcribed verbatim. The interviews will take 30 to 40 min. Interviews will continue until saturation is achieved (no new emerging facts or information) [43,44]. The data collection tool will be developed by the researchers.

Pregnant women will be interviewed to uncover the underlying cause of their activeness, anxieties, and strategies to increase their prenatal activity level [38]. We will analyze the interviews using a case study technique, which allows for multiple analyses of bound systems (cases) [38]. This technique involves collecting detailed data through interviews, reports, case descriptions, and case-based themes [45,46]. The coders will encode and recode the data based on the themes identified. The coders will create themes based on the objectives of the study and then use them to create categories for text message prototypes. Interviewers will ask the following questions:

- Do you think that exercise is beneficial to both mother and child and why?
- Do you think that prenatal physical activity and exercise during pregnancy reduce the risk of preterm labor, anxiety, enhance oxygen supply for the baby, and enable easy labor, and why?
- What strategies do you think can best increase your prenatal physical activity participation?

### 2.3. Phase 2: Assessment of Prototype Prenatal Messaging

During Phase 2, prototype text messages will be developed for each of the categories identified in Phase 1. Prototype text messages will provide mobile-friendly access to the MomConnect network and will be limited to 150 characters or fewer. Next, we will conduct interviews to ascertain the perceived preference and acceptability of the prenatal physical SMSs within the context of the setting [38]. A convenient sample of prenatal care providers, including physicians, nurses/midwives, and sedentary pregnant women who do not meet the recommended 150 min of moderate-intensity physical activity per week, will be interviewed. Participants in the interviews will be recruited in health facilities. Participants must be (i) a doctor, nurse/midwife, or pregnant patient at antenatal health facility, (ii) able to speak English and isiXhosa, and (iii) at least 18 years old to be eligible for the study. In-person interviews in a quiet designated place provided in each of the health facilities by the facility manager. Participants will be provided with three prototype text messages for each developing category. The interviewer will present the text messages to the participants and then ask the following questions [38]:

- Would you be able to share your perspective on the text messages?
- Do you believe these messages would support women in increasing their physical activity?
- What improvements do you believe could enhance the messages?
- Which SMS message, out of all of them, would you prefer, and why?

### 2.4. Phase 3: Users’ Preferred Messages Acceptable Dosage

Phase 3 will involve conducting a survey to ascertain the preferred and acceptable frequency of SMSs among 200 conveniently selected pregnant women for inclusion in the MomConnect content. The survey will include participants who are currently pregnant or have previously been pregnant, are 18 years and above, and can read and speak both English and IsiXhosa. We will recruit a convenience sample of pregnant women who are accessing antenatal health services from the Buffalo City Municipality in the Eastern Cape Province. The questionnaire will be self-administered to participants with comprehension or reading difficulties. We will determine the frequency (once per day, twice per day, once per week, once in two weeks, once per month) and precise times of day (morning: between 13:00 a.m.–12:00 p.m.; noon: between 12:00–16:00 p.m.; evening: between 16:00–22:00 p.m.) that women prefer to receive text messages regarding physical exercise during pregnancy [38].

### 2.5. Pilot Study

Prior to the implementation of a digital prenatal physical activity program utilizing SMS text messages for inclusion into the MomConnect health device to improve physical activity behavior during pregnancy, which is the expected outcome measure of this study, a pilot study of 20 reproductive-age women will be conducted to determine the comprehension and effectiveness of the prenatal physical activity SMS prototypes. The criteria for selection in the pilot study will be women who are aged 18–45 years, able to speak English and isiXhosa, and engaged in at least 30 min of moderate activity five days a week (more than 150 min) in the preceding week (self-reported) [38]. The participants in this phase will be asked to indicate whether they understand the relevance (content), preferred timing, and frequency of the prenatal physical activity SMS prototypes. The feedback from the outcome of the pilot study will be used to restructure the content, timing, and frequency of the prenatal physical activity SMS prototypes.

### 2.6. Recruitment Potential Challenges

This study may face recruitment challenges such as privacy and confidentiality concerns, technological barriers, and fear of stigmatization. These challenges will be addressed through clear explanations of data collection, storage, and usage, provision of written and verbal informed consent in a comprehensible language for the participants, engagement in culturally sensitive communication to respect the participants’ values and beliefs, and implementation of anonymization techniques alongside secure data storage methods.

### 2.7. Data Analysis

We will perform qualitative analysis using the thematic content analysis approach for Phases I and II, based on the qualitative interview data from the pregnant/non-pregnant women and the healthcare providers (physicians, midwives, and nurses). The individual interview transcripts will be analyzed using Atlas.ti 12.0. Descriptive analysis (frequency, percentages, mean, and standard deviation) will be applied to the quantitative data to summarize the variables related to the frequency of the SMSs, whether daily, twice daily, or weekly, and the timing of the test messages (time per day/number per trimester). The data will be analyzed using SPSS version 30.0.

### 2.8. Ethical Considerations

The Human Research Ethics Committee at the University of Fort Hare (Ref #2024=02=05 Okafor UB) approved the study protocol. Informed consent will be obtained from all participants before collecting data. Participants will be notified of their voluntary involvement and that they may withdraw at any time without prejudice. Furthermore, the participants’ confidentiality and privacy will be protected throughout. Only the researchers will have access to the recorded interviews. The researchers will conduct, analyze, and store both the qualitative and quantitative data in a cabinet until the completion of the research project using an interview guide. Similarly, the participant’s identity and information will remain hidden. Only the researchers will have access to the password-protected computer that has the data.

## 3. Discussion

The MomConnect device is devoid of physical activity and exercise content that would encourage physical activity during pregnancy. Therefore, this protocol outlines a research roadmap that outlines the steps involved in designing and developing a preferred prototype for prenatal physical activity and exercise text massaging. The developed prototype could potentially be integrated into the MomConnect technological health device to encourage women in the Eastern Cape Province of South Africa to engage in physical activity and exercise during pregnancy. This protocol study makes a unique contribution as the first to provide insights for designing physical activity and exercise content for the MomConnect device. The lack of awareness regarding prenatal physical activity and exercise constitutes a significant obstacle to physical activity engagement among women [20,23,24]. Consequently, enhancing women’s understanding of prenatal physical activity may significantly influence the adoption of such behavior. Engagement in prenatal physical activity can enhance clinical and maternal–child health outcomes [1,4,5,6,7,8,9,10,47]. Hence, efforts to encourage women’s physical activeness during and after pregnancy are crucial. The use of SMS messaging has the potential to create awareness and knowledge about prenatal physical activity and exercise.

Mobile health (mHealth) interventions demonstrate potential in enhancing maternal health outcomes, especially in resource-constrained settings. These interventions can improve access to maternal health information, promote health-seeking behaviors, and assist healthcare workers. A previous study in South Africa showed that text-message-based maternal mHealth interventions could provide pregnant women with timely, relevant, useful, and supportive information on birth preparedness and postnatal care [48]. Studies have shown that text messages encourage pregnant women to engage in more physical activity [37,49]. The interplay between content relevance and frequency may influence the effectiveness of text messages in promoting physical activity, with frequent irrelevant messages potentially reducing activity levels [50]. Irrelevant communication frequency can have a negative influence on physical activity, emphasizing the risk of providing irrelevant content in digital health strategies [50]. Therefore, contextually relevant prenatal physical activity SMS content could potentially be effective in encouraging physical activity among pregnant women when integrated into the MomConnect technological device. Once we have developed and designed the desired physical activity SMS specifically for pregnant women in this geographical setting, our study will provide the foundation to evaluate such a postulation. This study will emphasize the potential of mobile technologies to enhance physical activity among pregnant and postnatal women while also addressing the challenges associated with recruitment and sustaining engagement.

The findings of this study will assist in ascertaining the preferred dose of SMS messaging to promote prenatal physical activity and exercise among women. Similarly, the results of this study will provide the foundation for designing a randomized control trial to evaluate the effectiveness, efficacy, and acceptability of MomConnect in promoting prenatal physical activity among pregnant women within the framework of BCCM. If the MomConnect interventional approach proves effective, it could lead to a large-scale implementation among reproductive-age women in the province and across the country. Such information is necessary for strategic planning regarding the delivery of prenatal physical activity healthcare services. Finally, the findings of this study will assist policymakers in comprehending the significance of developing an innovative, low-cost intervention strategy to enhance prenatal physical activity and exercise, a frequently overlooked yet crucial aspect of antenatal healthcare.

## 4. Disseminating Findings

We will present and submit this study’s findings to the Eastern Cape Department of Health and key stakeholders. The key stakeholders will include maternity health team managers and obstetrics health providers (physicians, midwives, and nurses). We will present the findings at national and international conferences and publish them in accredited peer-reviewed national and international journals. In addition, we will publish a policy brief and an infogram to attract the attention of a non-academic audience. Similarly, we will disseminate the study’s findings through social media platforms like Facebook, Twitter, and Instagram.

## Figures and Tables

**Table 1:** Methodological summary of the protocol stratified by study phases.

Phase	Objective	Design	Population and Sample	Analysis	Outcome Measure
I	Conduct a scoping review on prenatal physical activity test SMS preferences and acceptability among pregnant women	Systematic Reviews and Meta-analyses (PRISMA) methodology for scoping review	Published articles meeting the selection criteria	Narrative synthesis	Evidence based on MomConnect’s effectiveness, efficiency, and acceptability as a facilitator of health behavioral outcomes
	Conduct formative research to determine a prenatal physical activity test SMS acceptable to pregnant women	Qualitative (interviews)	Pregnant women (purposive)	Case study approach	Content case-based themes of text message prototype development
II	Develop prototype prenatal physical activity text messages	Qualitative (interviews)	Prenatal care health providers (physicians, nurses/midwives) and pregnant women	Thematic content analysis	Feasibility and appropriateness of the prenatal physical activity test messages
III	Determine the acceptable dose of prenatal physical activity test messages	Quantitative (cross-sectional survey)	Pregnant and non-pregnant women	Descriptive analysis (frequency and percentages)	Acceptability and preferred prenatal physical activity SMSs desirable for inclusion in MomConnect device

## Data Availability

The original contributions presented in this study are included in the article. Further inquiries can be directed to the corresponding author.
